# Sound Interferes with the Early Tactile Manual Abilities of Preterm Infants

**DOI:** 10.1038/srep23329

**Published:** 2016-03-18

**Authors:** Fleur Lejeune, Johanna Parra, Frédérique Berne-Audéoud, Leïla Marcus, Koviljka Barisnikov, Edouard Gentaz, Thierry Debillon

**Affiliations:** 1 Child Clinical Neuropsychology Unit, FPSE, University of Geneva, Switzerland; 2 Intensive and Regular Neonatal Care Unit, CHRU Grenoble, France; 3 Sensorimotor, Affective and Social Development Unit, FPSE, University of Geneva, Switzerland; 4 University Grenoble Alpes, LPNC and CNRS, Grenoble, France

## Abstract

Premature birth is a sudden change of the sensory environment of a newborn, while their senses are still in development, especially in the stressful and noisy environment of the NICU. The study aimed to evaluate the effect of noise on the early tactile manual abilities of preterm infants (between 29 and 35 weeks PCA). Infants were randomly assigned to one of the two conditions: Silence and Noise. For each condition, two phases were introduced: a habituation phase (repeated presentation of the same object, prism or cylinder), followed by a test phase (presentation of the familiar or a novel object). In the Silence condition, they received the tactile habituation and test phases: In the Noise condition, they went through the same phases, while an alarm sounded. Sixty-three preterm infants were included. They displayed a strong and effective ability to memorize tactile manual information and to detect the difference between two shape features, but this ability seems to be impaired by the concomitant exposure to an alarm sound. This study is the first to highlight the effect of a negative stimulus on sensory functioning in premature infants. It reinforces the importance of developing environmental measures to lower the sound level in NICUs.

It has been widely documented that children born prematurely, before 37 weeks of gestation are at risk for long-term neurodevelopmental difficulties. This is both due to prematurity itself as well as an adverse postnatal environment^1^. A better understanding of the influence of the early exposure to a hospital environment on the sensory abilities of preterm infants, could help to adapt their sensory environment and stimulations in the neonatal period to improve their neurologic development.

Premature birth is a sudden change to a newborn whose environment was till then protected with little tactile stimulation, muffled sounds and soft lighting. The neonatal intensive care unit (NICU) is instead a noisy and bright place where painful tactile stimulations are sometimes common^2^. The environmental conditions of the NICU can lead to behavioral and physiological stress responses^3^ which can affect the immature nervous system of the preterm infant^1^,^4^,^5^. The impact of the NICU on cerebral development has also been noted by research on developmental care (i.e. Neonatal Individualized Developmental Care and Assessment Program), which aims to minimize neonatal distress by controlling the sensory environment. This has shown that developmental care has positive effects on brain development^6^. Numerous studies have evaluated the impact of noise on the physiological constants of the preterm newborns but few studies have analyzed the impact on their sensory development^7^.

Regarding auditory development, the structural components of the inner ear are present from the 15^th^ week of gestation but cochlear function becomes effective after 24 weeks^8^,^9^. It has been shown in animals that repeated exposure to sounds at high frequency altered the formation of neuronal connections in the central auditory nervous system^10^,^11^. Previous studies have found that the acoustic environment of the NICU contained an important amount of high frequency sounds (>500 Hz, corresponding to a high pitch sound)^12^. Moreover, most of the noise levels recorded in NICUs exceeded recommendations (45 dBA) from the American Academy of Pediatrics^13^. Thus, exposing premature infants to loud high frequency sounds, while the auditory system is still immature, could alter the normal development of hearing and induce language and attention disorders^14^. However, no study has investigated if and how the sounds could interfere with other sensory modalities, such as touch.

Tactile receptors are present from the 7^th^ week of pregnancy and develop all over the body by cephalo-caudal maturation until the 20^th^ week of gestation^15^. Grasping at birth is well-known as a reflex in response to a stimulation of the palm of the hand. This grasping is, however, not only a pure reflex. Recent studies analyzing the tactile manual abilities of premature newborns, have revealed that they were capable of memorizing tactile information of specific objects (prism and cylinder) and detecting the difference between two forms with either the right or left hand^16^. These abilities have been observed from the post-conceptional age of 28 weeks^17^. Preterm infants were also able to learn an object shape with one hand and discriminate a new-shaped object in the opposite hand^18^. Thus, tactile manual abilities seem to be an effective and strong learning process in preterm newborns. One can wonder if the presence of a high frequency sound to which preterm infants are exposed on a daily basis could modify the effectiveness of the tactile sensory process.

Research in full-term newborns have revealed essential insights related to early sensory integration^19^. For example, newborns are able to transfer shape information from touch to vision^20^. They can also recognize a familiar face only when they are previously habituated with the speaking face, but not when the speech sounds are removed^21^. They responded to abstract numerical quantities across visual and auditory modalities^22^. These studies showed an early functional communication between different sensory modalities, allowing us to hypothesize that the presentation of a sound could have an impact on tactile manual abilities.

The main objectives of this study were to evaluate the relation between a sound introduced daily (in this case the alarm of the enteral feeding pump) and (1) the early tactile manual habituation abilities, and (2) the early tactile manual discrimination abilities of preterm infants. The alarm sound was expected to make the habituation and discrimination processes more difficult.

## Results

### Participants

Sixty-three preterm infants (31 girls and 32 boys) were included in the study: 26 in the Silence condition and 37 in the Noise condition. An additional 22 preterm infants were excluded from the study after one trial because of drowsiness (seven), sleep (two), fussing (five), or technical problems (eight). Regarding the state of arousal (drowsiness, sleep, fussing), 6 preterm infants were part of the Noise condition and 8 preterm infants were part of the Silence condition. Moreover, the hearing was verified retrospectively by collecting the data of automated auditory evoked potentials, measured before hospital discharge by a specialist, and none had hearing impairment.

### Habituation phase

**Figure 1:**
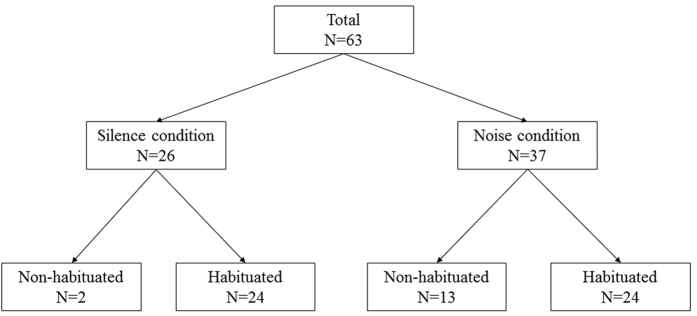
Flow chart of participants in the study.

Firstly, 15 preterm infants within the whole population did not reach the habituation criterion after 10 trials and were thus considered as being not habituated (Fig. 1). In previous studies, all preterm infants attained this criterion before the 10^th^ trial, so we examined this unexpected result further. We compared the proportion of habituated and non-habituated infants in each condition: more infants failed to habituate in the Noise (35.1%) condition than in the Silence (7.7%) condition (*χ*^*2*^(1) = 6.34, *p* = 0.012). Moreover, general and medical characteristics of the Noise condition did not differ significantly between the non-habituated and habituated infants (all *p* > 0.05). Results are presented in Table 1.

Secondly, 48 preterm infants attained the criterion for habituation before the 10^th^ trial: half of the infants were part of the Noise condition, and the other half of the Silence condition. Information regarding general and medical characteristics can be seen in Table 2. No significant difference between the two conditions was found.

The parameters of habituation for the Silence and the Noise conditions are reported in Table 3. Results revealed a significant effect of the condition for the total holding time occurring until the habituation criterion was reached *(p* = 0.024, *η*^*2*^_*p*_ = 0.11) and for the mean number of trials conducted *(p* = 0.007, *η*^*2*^_*p*_ = 0.15). Preterm infants in the Noise condition needed more time and trials to attain the habituation criterion than in the Silence condition. No significant effect of the condition was found for the total holding times for the first 2 trials (*p* = 0.48, *η*^*2*^_*p*_ = 0.01). There were no other significant effects for the shape factor, and the condition × shape interaction (all *p* > 0.05).

Finally, we compared habituation parameters between the control and experimental groups. Student’s t tests revealed no significant differences between the two groups (all *p* > 0.05).

### Test phase

**Figure 2:**
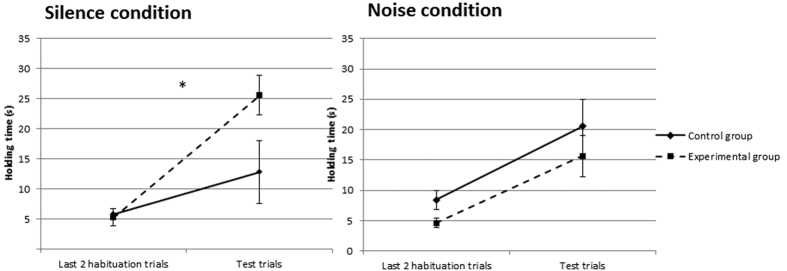
Means and standard errors of discrimination according to the conditions (Silence vs. Noise) and the group (Control/familiar object vs. Experimental/novel object).

Figure 2 illustrates the results of the discrimination process in the 2 conditions, according to the groups.

#### Silence condition

The results showed a significant effect of the phase factor *(p* < 0.001, *η*^*2*^_*p*_ = 0.5), as well as a significant phase × group interaction *(p* = 0.031, *η*^*2*^_*p*_ = 0.19). No significant effect of the group factor was found *(p* = 0.094, *η*^*2*^_*p*_ = 0.12). Planned comparisons revealed that the Experimental group held the novel object significantly longer than in the last two habituation trials (*p* < 0.001, *η*^*2*^_*p*_ = 0.53). However, in the Control group, mean holding time for the familiar object and mean holding time for the last 2 habituation trials did not differ significantly (*p* = 0.10, *η*^*2*^_*p*_ = 0.12).

#### Noise condition

Results revealed a significant effect of the phase factor *(p* < 0.001, *η*^*2*^_*p*_ = 0.44): preterm infants held the objects significantly longer during the 2 test trials than during the last 2 habituation trials. No significant effect of the group factor was found *(p* = 0.17, *η*^*2*^_*p*_ = 0.08), nor was there any significant phase × group interaction (*p* = 0.84, *η*^*2*^_*p*_ = 0.002).

## Discussion

This study highlights for the first time the detrimental influence of an unpleasant common sound on sensory learning in preterm newborns. More precisely, our results reveal the negative impact of the alarm sound of an enteral feeding pump on their early manual abilities.

First, a significantly higher proportion of preterm infants failed to habituate to the object after ten trials when they were exposed simultaneously to the alarm sound. These infants seem to have important difficulties to deal with the tactile learning when the noise is present. This interpretation is reinforced by the result of this study related to the Silence condition (discussed below). Indeed, preterm infants who succeeded to habituate to the object in the Noise condition, needed more time and trials to attain the habituation criterion than those in the Silence condition. Consistent with previous studies, preterm infants displayed a strong and effective tactile habituation process in the Silence condition^17^,^18^,^23^. However, this ability to memorize tactile information seems to be weakened by the concomitant exposure to the alarm sound of the feeding system.

Secondly, the infants of the Silence condition held the novel object, presented during the test phase, significantly longer than the familiar one during the last two habituation trials, confirming the presence of manual discrimination between a prism and a cylinder in preterm infants^17^,^18^,^23^. This result was objectivized by the control group, who were presented with the same object throughout the entire experiment and who displayed, as expected, an equivalent holding time between the end of the habituation and the test phase. In contrast, in the presence of the alarm sound, the experimental group, but also the control group, held the objects significantly longer during the two test trials than during the last two habituation trials. These results question the effectiveness of the tactile habituation process in the Noise condition. It is likely that the alarm sound has a deleterious effect on the learning process of habituation and compromises the ability of preterm infants to discriminate shape features. The exposure to the noise seems thus to alter especially the memorization of the previous tactile manual experience.

In our study, no statistical difference was found in gender, gestational age, post-natal age, post-conceptional age, birth weight, weight at test and medical characteristics according to the condition. Consequently, the difference of results between the two conditions could not be explained by the degree of immaturity, nor by the medical history of these infants.

Multisensory integration is fundamental for the understanding of our environment^24^. All senses are interconnected in a complex way and brain imaging studies have shown that cortical pathways were not specific to one sense but could be modulated by signals from other sensory modalities^25^,^26^,^27^. Our results reveal an early functional communication between tactile and auditory modalities in preterm infants. Numerous studies in adults and animals have shown that the combination of two sensory modalities could increase acuity in one modality and facilitate behavioral benefits^28^,^29^,^30^ whereas excessive stimulation of one sense (i.e. visual) could alter not only visual functioning but also auditory and intersensory functioning^31^,^32^. In our study, the alarm sound activated the auditory modality, which interfered with the attentional resources necessary in the tactile modality for manual habituation and discrimination processes.

Numerous studies have focused on the detrimental effect of noise on the physiological constants of the newborn. From the 26^th^ gestational week, intense sound causes a change in heart rate, blood pressure, respiratory rate and oxygenation^33^. Indeed, it was shown that noise had effects on blood pressure^34^, breathing^35^,^36^,^37^, and disrupted sleep^38^ which may be harmful for their neurological development^39^. Furthermore, excessive noise is suspected to influence the neuroendocrine and immune system^7^,^33^. These studies suggested that intense sound acts as a stressful event on physiological self-regulatory skills. Our results reveal for the first time that the disorganization created by the alarm sound also altered the sensory functioning in preterm infants.

The standards recommended for NICUs were updated by the American Academy of Pediatrics in 2007^40^. It is stipulated that inside the room, but outside the incubator, the maximum noise level must be less than 45 dBA. A previous study has shown that sounds generated inside the mother (i.e. maternal respiratory, intestinal activity, etc.), in the absence of externally sounds, was as low as 50 dB above 200 Hz (corresponding to 40 dBA)^41^, confirming the validity of this recommendation. In our study, the noise level generated by the feeding pump and the one recorded outside the incubator (55 to 63 dBA) were above the standards recommended. Moreover, the alarm is a high frequency sound (2450 Hz) which could alter the formation of neuronal connections in the central auditory nervous system^10^,^11^. Despite sound levels above the one recommended and its high frequency, we chose to maintain this sound source to mimic the real condition of everyday life. In the NICU, preterm infants are exposed to that noise a minimum of 8 times per day, and that is not counting the other alarms that may be present. In most studies, 65 dBA was considered as harmful noise level^42^,^43^,^44^,^45^. Moreover, intermittent auditory stimulation in the NICU can provoke a pain-like stress response^46^ that can be responsible of behavioral inhibition^47^ and decreased abilities to adjust to environmental stimuli^48^. Furthermore, excessive noise affects the functional organization of the developing auditory cortex^49^,^50^. At noise levels higher than those of the standards recommended, we have found that preterm infants had their tactile manual abilities disturbed.

The neonatal period is a critical period when neural circuitry is first generated^51^ and it is now known that cerebral plasticity and cognitive development is intimately linked with early sensory experience^52^,^53^,^54^. Individualized developmental care aim to minimize discomfort by controlling neonatal sensory stimuli and the beneficial impact of this care on the developing brain has already been proven^6^. The alarm of the enteral feeding system, used in our study, sounds when the syringe is almost empty, and sounds again when the syringe is empty. The alarm’s usefulness is far from vital to the survival of the newborn and this alarm should thus be removed from the NICU. Overall, this study confirms the importance of developing environmental measures to lower the sound level in NICUs as the establishment of light alarms^55^, architectural modifications, sound absorbing materials, minimizing loud staff conversation, optimizing choice of equipment^56^,^57^,^58^ and promoting individualized developmental care. These measures will protect the early sensory functioning of preterm infants, which in turn could improve their long-term neurodevelopmental outcome.

Limitations to the generalizability of our findings should be acknowledged. One of the limits of this study is the impossibility of being realized in blind conditions. Another limit is the impossibility to control all sensorial modalities such as smell or vision, even if we hid the wooden object from the newborn’s sight. Finally, it would have been interesting to compare the two conditions (Silence and Noise) for the same infant in order to control for the important inter-individual differences existing in this population. However, we decided to compare different infants in each group as it was already difficult to obtain one stable and valid quiet awakening state for each preterm infant, as well as to avoid possible effect of tactile learning between the two conditions.

In conclusion, premature birth involves an abrupt change in the sensory environment of the newborn while all their senses are still developing. This study is the first to highlight the effect of a negative stimulus on sensory functioning in premature infants. Moreover, it brings new insights for supporting developmental care by confirming the importance to lower the sound level in NICUs, and avoid the use of unpleasant and non-vital sounds such as the alarm of the enteral feeding system.

## Methods

This study was prospective, observational and carried out between March 2014 and April 2015. Parents gave written consent for their baby to participate to the experiment. The present study was conducted in accordance with the Declaration of Helsinki and approved by the ethics committee of the FPSE of the University of Geneva.

### Participants

Participants were preterm infants born between 24 and 34 weeks of gestation and assessed between 29 and 35 post-conceptional age. The study site was a level III NICU in Grenoble Hospital (France), which includes 26 private rooms containing between one and three beds. We excluded from the study preterm infants with a polymalformative syndrome, those with cystic periventricular leukomalacia or grade III or IV intraventricular haemorrhage based on their cranial ultrasound, and those who received sedatives or anticonvulsive treatment during the experiment. For organizational reasons, the experiment took place two days per month so the recruitment was opportunistic, considering the inclusion and exclusion criteria.

Sample size was determined according to a power analysis (using Gpower) based on a 2 × 2 mixed interaction with an α error probability at 0.05, a power at 0.95 and an effect size at 0.45 suggesting a total sample size of 50 participants.

### Stimuli

The stimuli consisted of a cylinder (a smoothly curved shape, 35 mm long and 6 mm in diameter) and a prism (a sharply angled shape, 9 × 6 × 6 mm triangle base). These objects were identical to those used in previous studies^17^,^18^,^23^. The experiment consisted in a tactile stimulation of the left hand, as it was mostly free of any prosthesis and no hand differences have been found in previous studies^17^,^19^.

The syringe pump was a pilot A2 from the FRESENIUS firm and was placed on the right side of the incubator (opposite to the opened side – tested hand). The sound level was recorded using a sound dosimeter. The alarm produced a mean 63 dBA noise outside the incubator and 55 dBA noise inside the incubator, and its frequency was 2450 Hz. To control the other sounds of the NICU, the experimenters turned off the alarms from other medical equipment. Furthermore, if other people (medical staff or parents) were present in the room, they were asked to remain silent.

### Procedure

Each infant was swaddled comfortably and assessed in his incubator just before or just after care, in an arousal state of 4 on the Brazelton scale (quiet wakefulness)^59^, and more than 1 hour after being fed. This experiment was run without visual control: the experimenter positioned the infant’s head in the opposite side of the tested hand. The whole experiment was videotaped for subsequent analysis. It was conducted in two phases: the habituation phase, then the test phase for the habituated babies.

#### The habituation phase

The habituation consisted of the repeated presentation of a wooden object (prism or cylinder), in successive trials. Infants were randomly assigned to the Silence condition or the Noise condition (a random number table computer-generated was used for randomization). In the Noise condition, the first experimenter activated the alarm of the syringe pump. For the two conditions, the second experimenter placed an object in the infant’s left hand. Infants were equally randomized to receive the prism or the cylinder. When the infant grasped the object, the first experimenter started recording the holding time. The recording was stopped when the infant released the object, after having held it for at least 1 second, marking the end of the trial. If the infant held the object for longer than 60 seconds, the second experimenter gently opened the infant’s hand and removed the object. Then, she immediately presented the object again, beginning a new trial. Habituation trials continued until the duration of holding in any 2 consecutive trials, from the third trial onward, totalled a third or less of the total holding time in the first 2 trials. If the habituation criterion was not met by the 10^th^ trial, the experiment was stopped and the infant was considered as being not habituated.

#### The test phase

The test phase was conducted immediately after a successful habituation, and was composed of 2 test trials. Half of the infants (Control group) were presented with the familiar object, and the other half (Experimental group) were given the new-shaped object. In the Noise condition, the first experimenter turned off the alarm of the syringe pump at the end of the experiment.

### Data collection

All relevant general and medical characteristics from the medical records of infants were collected. Three parameters of habituation were measured: mean total holding times occurring until the habituation criterion was reached, total holding times for the first 2 trials, and mean number of trials conducted. The mean holding time for the 2 test trials was also calculated.

### Statistical analysis

All statistical analyses were conducted using SPSS 22.0 (IBM SPSS Statistics, IBM Corporation). Pearson’s chi-square or Fisher’s exact test was used for the comparison of qualitative variables. Analysis of variance (ANOVA) was performed for each parameter of habituation, with “condition” (silence vs. noise) and “shape” (prism vs. cylinder) as 2 between-subjects factors. The discrimination process was then examined by performing a repeated-measure ANOVA for each condition, with “phase” as a within-subjects factor (last 2 habituation trials vs. discrimination trials) and “group” as a between-subjects factor (control vs. experimental). The significant threshold was 0.05.

## Additional Information

**How to cite this article**: Lejeune, F. *et al.* Sound Interferes with the Early Tactile Manual Abilities of Preterm Infants. *Sci. Rep.* **6**, 23329; doi: 10.1038/srep23329 (2016).

## Figures and Tables

**Table 1:** Preterm infants’ general and medical characteristics according to the failure (non-habituated) or success (habituated) to attain the habituation criterion.

Characteristics	Habituated (N = 48)	Non-habituated (N = 15)	p
	n (%) or mean (SD)	n (%) or mean (SD)	
Condition			**0.012**
silence	24 (92.3)	2 (7.7)	
noise	24 (64.9)	13 (35.1)	
Gender			0.34
girl	22 (45.8)	9 (60)	
boy	26 (54.2)	6 (40)	
Gestational age (weeks)	30 + 1 (3)	30 + 5 (2.3)	0.50
Post-natal age (days)	19.7 (17.6)	17.3 (14.4)	0.63
Post-conceptional age (weeks)	33 (1.6)	33 + 1 (1.3)	0.61
Birth weight (g)	1351 (478)	1319 (432)	0.82
Weight at test (g)	1608 (323)	1541 (368)	0.50
Antenatal steroids treatment	44 (91.7)	15 (100)	0.25
SGA	11 (22.9)	6 (40)	0.19
Caesarean delivery	27 (56.3)	11 (73.3)	0.24
Apgar score <7 at 5 min	4 (8.3)	4 (26.7)	0.07
Apgar score <7 at 10 min	2 (4.2)	1 (6.7)	0.69
Intubation	18 (37.5)	7 (46.7)	0.53
nCPAP during hospitalization	36 (75)	10 (66.7)	0.53
nCPAP during the test	13 (27.1)	5 (33.3)	0.64
Nasal cannula	29 (60.4)	10 (66.7)	0.66
Central venous catheter	39 (81.3)	13 (86.7)	0.63

Note: nCPAP, nasal continuous positive airway pressure; SGA, Small for Gestational Age.

**Table 2:** Habituated preterm infants’ general and medical characteristics according to the conditions.

Characteristics	Silence condition (N = 24)	Noise condition (N = 24)	p
	n (%) or mean (SD)	n (%) or mean (SD)	
Gender			0.86
Girl	12 (50)	10 (41.7)	
Boy	12 (50)	14 (58.3)	
Gestational age (weeks)	30 + 1 (2.8)	30 (3.2)	0.84
Post-natal age (days)	19.5 (15)	19.9 (20)	0.94
Post-conceptional age (weeks)	33 (1.6)	32 + 6 (1.6)	0.84
Birth weight (g)	1415 (475)	1288 (483)	0.37
Weight at test (g)	1675 (329)	1541 (309)	0.15
Antenatal steroids treatment	23 (95.8)	21 (87.5)	0.86
SGA	4 (16.7)	7 (29.2)	0.47
Caesarean delivery	12 (50)	15 (62.5)	0.83
Apgar score <7 at 5 min	3 (12.5)	1 (4.2)	0.06
Apgar score <7 at 10 min	1 (4.2)	1 (4.2)	0.18
Intubation	11 (45.8)	7 (29.2)	0.45
nCPAP during hospitalization	18 (75)	18 (75)	0.74
nCPAP during the test	4 (16.7)	9 (37.5)	0.10
Nasal cannula	16 (66.7)	13 (54.2)	0.86
Central venous catheter	20 (83.3)	19 (79.2)	0.93

Note: nCPAP, nasal continuous positive airway pressure; SGA, Small for Gestational Age.

**Table 3:** Parameters of habituation (total holding times, holding times for the first 2 trials, and number of trials during habituation) according to the object shape and the condition; Mean (standard deviation).

Habituation parameters	Silence condition			Noise condition			p
	Total	Prism	Cylinder	Total	Prism	Cylinder	
Total holding time (s)	106.3 (64)	107.7 (71)	104.9 (61)	158.5 (86)	159.2 (65)	157.8 (108)	**0.024**
First 2 trials (s)	59.7 (39)	56.1 (35)	63.3 (45)	67.2 (30)	67.8 (29)	66.5 (32)	0.48
Number of trials	5.3 (1.2)	5.1 (1.1)	5.4 (1.4)	6.5 (1.7)	6.5 (1.6)	6.5 (2)	**0.007**
